# 545 Contrast and Similarities Between Self-Immolation and Self-Injury Burn Patients

**DOI:** 10.1093/jbcr/irad045.142

**Published:** 2023-05-15

**Authors:** Ashley Honea, Nathan Zhang, Claudia Islas, Karen Richey, Kevin Foster

**Affiliations:** AZ Burn Center at Valleywise Health, Phoenix, Arizona; AZ Burn Center at Valleywise Health, Phoenix, Arizona; AZ Burn Center at Valleywise Health, Phoenix, Arizona; Arizona Burn Center Valleywise Health, Phoenix, Arizona; AZ Burn Center at Valleywise Health, Phoenix, Arizona; AZ Burn Center at Valleywise Health, Phoenix, Arizona; AZ Burn Center at Valleywise Health, Phoenix, Arizona; AZ Burn Center at Valleywise Health, Phoenix, Arizona; Arizona Burn Center Valleywise Health, Phoenix, Arizona; AZ Burn Center at Valleywise Health, Phoenix, Arizona; AZ Burn Center at Valleywise Health, Phoenix, Arizona; AZ Burn Center at Valleywise Health, Phoenix, Arizona; AZ Burn Center at Valleywise Health, Phoenix, Arizona; Arizona Burn Center Valleywise Health, Phoenix, Arizona; AZ Burn Center at Valleywise Health, Phoenix, Arizona; AZ Burn Center at Valleywise Health, Phoenix, Arizona; AZ Burn Center at Valleywise Health, Phoenix, Arizona; AZ Burn Center at Valleywise Health, Phoenix, Arizona; Arizona Burn Center Valleywise Health, Phoenix, Arizona; AZ Burn Center at Valleywise Health, Phoenix, Arizona

## Abstract

**Introduction:**

Self-immolation is an uncommon method of attempted suicide involving flammable substances. By contrast self-inflicted burn injuries utilize a chemical or heated object to cause injury, without suicidal intent. Both groups require psychological care post-hospitalization, but the successful rehabilitation of these populations may be compromised by availability of mental health services, patient resources and patient specific factors. The purpose of this study was to compare and contrast the two groups and evaluate post-discharge success.

**Methods:**

This was a retrospective chart review of patients admitted over a 7-year period with either self-immolation (SIM) or self-inflicted (SIF) burn injury. Prospectively, patients were called to evaluate current status using the SF12 questionnaire. Basic descriptive statistics were calculated.

**Results:**

A total of 90 patients met inclusion/exclusion criteria, 55 SIM and 35 SIF. Both groups were predominantly white males, and the average age was SIM=36.4 vs SIF=37.9 years (p=0.668). Flame was the most common mechanism of injury for both groups, 93% of SIM used an accelerant vs 26% of SIF (p< .00001). While overall, home was the most common location for the event to occur for both groups, n=19 SIM and n=17 SIF, 17 of SIM occurred in a public place vs 4 of SIF (p=.033). Both groups SIM vs SIF, while not significantly different, had high rates of substance use 75% vs 63%, serious mental illness 42% vs 46%, Title 19 support 29% vs 26% and prior suicide attempts 62% vs 51%. There were no significant differences between groups for psychiatric history or number of individual psychiatric diagnosis/patient (µ 1.53 vs 1.54). Significant differences were found between the SIM and SIF groups for TBSA 46.1% vs 10% (p< .00001), prior involvement of a psychiatric case manager 12 vs 0 (p=.003) and mortality 22 vs. 0 deaths (p< .00001). Contact was successful for 5 SIM with 3 agreeing to take survey, for the SIF group 1 was able to be reached and declined the survey. The average score for SF12 physical domain was 43.06 and 44.14 for the mental domain, both below the national average of 50 points.

**Conclusions:**

The results of this study demonstrate the premorbid psychiatric conditions in this patient population. The major differences between groups were the degree of injury and mortality. Our inability to contact patients may be indicative of ongoing social instability. There remains a significant knowledge gap, indicating the need for prospective research to identify the most successful combination of therapies to both prevent and treat these injuries.

**Applicability of Research to Practice:**

This study highlights the need for a risk assessment tool to identify high risk groups and an integrated approach in the outpatient burn arena with dedicated behavioral health clinicians.

